# Cost-effectiveness analysis of artificial intelligence-assisted risk stratification of indeterminate pulmonary nodules

**DOI:** 10.1371/journal.pone.0343492

**Published:** 2026-03-05

**Authors:** Caroline M. Godfrey, Ashley A. Leech, Kevin C. McGann, Jinyi Zhu, Hannah N. Marmor, Sophia Pena, Lyndsey C. Pickup, Fabien Maldonado, Evan C. Osmundson, Stacie B. Dusetzina, Eric L. Grogan, Stephen A. Deppen

**Affiliations:** 1 Department of Surgery, Vanderbilt University Medical Center, Nashville, Tennessee, United States of America; 2 Department of Health Policy, Vanderbilt University School of Medicine, Nashville, Tennessee, United States of America; 3 Department of Thoracic Surgery, Vanderbilt University Medical Center, Nashville, Tennessee, United States of America; 4 Optellum Ltd, Oxford, United Kingdom; 5 Division of Allergy, Pulmonary, and Critical Care Medicine, Vanderbilt University Medical Center, Nashville, Tennessee, United States of America; 6 Department of Radiation Oncology, Vanderbilt University Medical Center, Nashville, Tennessee, United States of America; 7 Tennessee Valley Healthcare System, Nashville, Tennessee, United States of America; Inha University Hospital, KOREA, REPUBLIC OF

## Abstract

**Background:**

Artificial intelligence-based radiomic approaches have been shown to accurately evaluate indeterminate pulmonary nodules. With the expansion of lung cancer screening and utilization of computed tomography imaging, indeterminate pulmonary nodules requiring diagnostic evaluation are increasingly common. Accurate non-invasive characterization may reduce time to cancer diagnosis and decrease invasive procedures for benign disease, but the cost-effectiveness of AI-based methods has not been quantified. We sought to evaluate the cost-effectiveness of AI-assisted clinician evaluation compared to clinician evaluation alone for the cancer risk stratification of patients with indeterminate pulmonary nodules.

**Methods:**

We constructed a decision model assuming guideline-based care from a payer perspective with a lifetime horizon. The base case is a 1.1 cm incidentally discovered IPN in a 60-year-old operative candidate in a clinical population with a 65% malignancy prevalence. Cost per life-year gained (LYG) was the primary outcome. We conducted deterministic sensitivity analyses on all parameters and performed a probabilistic sensitivity analysis. Given clinical variability of malignancy prevalence, we assessed the malignancy prevalence threshold at which utilization of AI would be cost-effective.

**Results:**

AI-supported clinician risk stratification resulted in an increase of 0.03 life years compared to clinician alone. With a 65% malignancy prevalence, AI was cost-effective with an incremental cost-effectiveness ratio (ICER) of $4,485/LYG. When the malignancy prevalence was < 5%, the ICER for AI support exceeded a standard willingness-to-pay threshold of $100,000/LYG.

**Conclusions:**

In clinical settings with a pre-test probability of malignancy exceeding 5%, AI-supported IPN risk stratification is cost-effective compared to clinician assessment alone.

## Introduction

Nearly 15 million Americans are now eligible for annual lung cancer screening with low dose computed tomography (CT) imaging following the 2021 expansion of lung cancer screening guidelines by the United States Preventive Services Task Force [1]. In addition to screening-identified pulmonary nodules, CT is increasingly used for medical diagnostic imaging, leading to a larger number of incidentally-identified pulmonary nodules [2]. This influx of indeterminate pulmonary nodules (IPN) represents a significant healthcare burden as IPNs require diagnostic evaluation to estimate the probability of lung cancer.

Currently, an an estimated 10–15% of surgical resections are performed for benign disease [3,4]. Though reducing the rate of non-therapeutic surgical resection is warranted, this must be balanced with the imperative to identify and diagnose lung cancer in its earliest, most treatable stage. Guidelines published by the American College of Chest Physicians recommend initial nodule stratification into low-probability (<5% probability of malignancy), intermediate-probability (5–65% probability of malignancy), and high-probability groups (>65% probability of malignancy) [5]. Correct classification of benign nodules into the low-probability group and malignant nodules into the high-probability group may reduce invasive diagnostic testing for benign nodules and reduce time to diagnosis for malignant nodules.

New biomarkers and clinical risk prediction models are being developed to aid in correct classification of IPNs into low- and high-probability groups. Radiomics is an emerging field which uses machine learning to model the probability of malignancy based on quantitative imaging features, usually on chest CT images.

The Lung Cancer Prediction Score (LCP Score) is part of an FDA-cleared software platform developed to aid clinicians in accurate lung nodule stratification. The underlying AI was trained and validated on a wide variety of data [6–8]. When tested in a 12-reader study, the accurate classification of lung nodules improved with LCP Scores as compared to clinicians reading alone [9].

Effective July 1^st^, 2022, the LCP Scores have a newly assigned common procedural terminology (CPT) code and is now eligible for reimbursement through the Centers for Medicare & Medicaid Services (CMS) at a cost range of $600–700 [10]. However, the cost effectiveness of LCP Scores for pulmonary nodule risk stratification has not yet been studied. We sought to compare the cost-effectiveness, measured in cost per life-year, of utilization of LCP Scores as a tool to improve pulmonary nodule risk stratification to clinicians reading alone by developing a model to simulate the subsequent clinical decision-making process and resulting health outcomes.

## Methods

### Model overview

**Fig 1:**
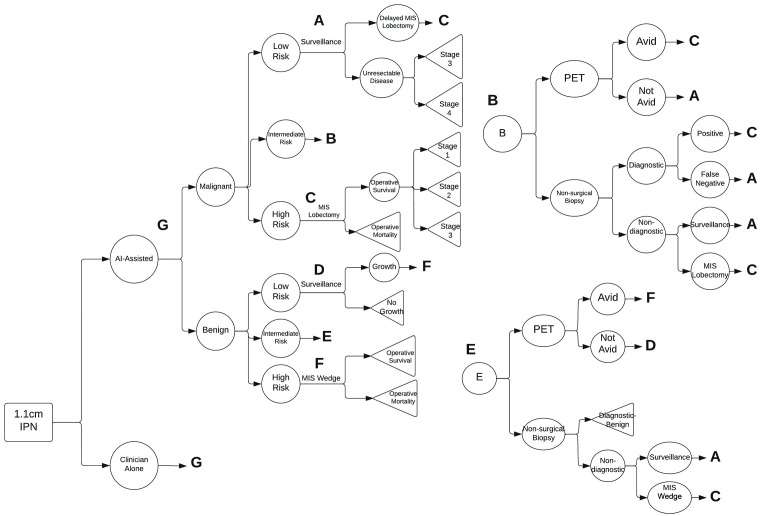
Decision Model Structure. The decision tree structure models the risk-stratification of an indeterminate pulmonary nodule utilizing artificial intelligence-assistance compared to the clinician alone. Repeated portions of the model have been collapsed into subtrees (A-G) for readability, each of which represents a diagnostic or management pathway that appears in various parts of the model (‘A’ = surveillance; ‘B’ = PET-CT evaluation; C = minimally invasive surgical (MIS) lobectomy; ‘D’ = low-risk surveillance; ‘E’ = intermediate-risk; ‘F’ = MIS wedge resection; ‘G’ = initial risk classification).

We constructed a decision analysis model to compare IPN stratification performed by the clinician alone to stratification performed by the clinician with AI support from the payer perspective with a lifetime horizon (Fig 1). The decision tree models subsequent care based on the risk stratification assuming guideline-based management. Outcomes included discounted life years and incremental cost-effectiveness ratios (ICERs).

The base case is a 1.1 cm incidentally-identified IPN in a 60-year-old patient who is a medically operative candidate. The selection of the base case parameters is meant to represent a standard patient evaluated in a pulmonology or thoracic surgery clinic. The base case assumed a malignancy prevalence of 65% to represent the malignancy prevalence observed in thoracic surgery or dedicated pulmonary nodule clinics, though this parameter was varied widely as detailed in sensitivity analyses [11].

We constructed the model using TreeAge Pro Healthcare (Version 2022 R2.0) software. The model stratified the patients to low/medium/high risk, either using clinician or clinician-plus-AI reasoning, and then followed the costs and LYG related to assigning either a benign lesion or a cancer in that way. Costs and benefits (life years) were discounted at a 3% annual discount rate. We converted all costs to 2021 United States Dollars (USD) using Gross Domestic Product Price Indices [12,13].

### Model structure

The model structure is a decision tree with costs and benefits assigned at each terminal node. Disease-specific costs were considered one-time costs. Unrelated medical costs accrued at each year of survival, considering the life years associated with each terminal node. We measured benefits in life years (derivation detailed in Outcomes section of Methods).

This decision tree models the initial choice of risk stratification method (AI-support versus clinician alone). The determination of the true malignancy status of the nodules (benign versus malignant) for patients is based on the malignancy prevalence within the population modeled. The decision tree subsequently incorporates probabilities of assigning patients to each cancer risk probability group based on data from the reader study that compared the clinician working alone to clinicians working with AI assistance [9]. Subsequent diagnostic steps followed clinical care consistent with the American College of Chest Physicians IPN evaluation and management guidelines [5].

### Model assumptions

All high-probability nodules were assumed to be anatomically amenable to wedge resection and underwent an initial diagnostic wedge resection with frozen section analysis. Upon confirmation of a benign diagnosis, we considered patients operatively complete. Upon confirmation of a malignant diagnosis, patients then underwent a definitive lobectomy in the same operation. Though anatomic segmentectomy is now acceptable for early-stage lung cancer, given that this practice is not widespread at this time, patients with lung cancer that underwent surgical resection received a lobectomy [14].

Patients with nodules demonstrating Positron Emission Tomography (PET) avidity subsequently received a surgical biopsy, while patients with nodules that did not show PET-avidity underwent surveillance. Additionally, in the case of patients receiving a non-surgical biopsy with a non-diagnostic result, patients in the model either underwent surgical biopsy or surveillance. We varied the probability of surgical biopsy from 0 to 1 in sensitivity analyses to account for practice variation.

### Model parameters

We preferentially used parameter values from meta-analyses and randomized controlled trials. If these preferred sources were not available, we used values from observational studies. When literature-derived values were not available, we substituted expert opinion or values utilized in prior cost-effectiveness analyses (Table 1). We derived risk stratification parameters from a 12-reader study previously published validating the LCP Score (Table 1) [9]. We calculated the frequency of expert reader classification into low-, intermediate-, and high-probability groups for the malignant and benign nodule groups included in the 12-reader study. The same was done for expert readers with the assistance of LCP Score. Next, we converted these values to probabilities based on the likelihood that a given malignant (or benign) nodule would be classified into each of the stated risk groups. The twelve expert readers in this study were composed of six pulmonologists (two with expertise in thoracic oncology) and 6 radiologists (two with expertise in thoracic radiology) described in further detail in the original reader study [9].

**Table 1:** Model Parameters.

Parameter	Base Case Parameter	Sensitivity Analysis Range	PSA distribution	References
***Probability Parameters***				
*Malignancy Prevalence*	0.65	.01−.8	Beta	[9]
*Lobectomy mortality*	0.022	.01−.03	Beta	[15]
*Wedge mortality*	0.0083	.005−.015	Beta	[16]
*Benign nodule growth*	0.1	.08−.12	Beta	[17]
*PET avid, malignant*	0.89	.85−.92	Beta	[18]
*PET avid, benign*	0.23	.19−.51	Beta	[18]
*Diagnostic biopsy, benign*	0.56	.45−.67	Beta	[19]
*Diagnostic biopsy, malignant*	0.92	.74-1.0	Beta	[19]
*False negative biopsy, malignant*	0.037	.03−.044	Beta	[19]
***Immediate MIS resection***				
*Stage I NSCLC*	0.75	.6−.82	Dirichlet	[13]
*Stage II NSCLC*	0.17	.14−.2	Dirichlet	[13]
*Stage III NSCLC*	0.08	*	Dirichlet	–
***Delayed MIS resection***				
*Stage I NSCLC, postoperative*	0.72	.58−.75	Dirichlet	[13]
*Stage II NSCLC, postoperative*	0.19	.15−.23	Dirichlet	[13]
*Stage III NSCLC, postoperative*	0.09	*	Dirichlet	–
*Progression to unresectable*	0.04	.02−.06	Beta	[13]
*Stage III NSCLC, unresectable*	0.5	.2−.8	Beta	[13]
*Stage IV NSCLC, unresectable*	0.5	*	Beta	–
***Risk Stratification Parameters***				
***Clinician alone, malignant***				
*High risk*	.47	.2−.58	Beta	[7]
*Intermediate risk*	.42	*		[7]
*Low risk*	.10	.01−.27	Beta	[7]
***Clinician alone, benign***				
*High risk*	.11	.02−.5	Beta	[7]
*Intermediate risk*	.39	*		[7]
*Low risk*	.50	.18−.71	Beta	[7]
***AI-assisted, malignant***				
*High risk*	.60	.45−.75	Beta	[7]
*Intermediate risk*	.35	*		[7]
*Low risk*	.05	.02−.08	Beta	[7]
***AI-assisted, benign***				
*High risk*	.09	.04−.15	Beta	[7]
*Intermediate risk*	.37	*		[7]
*Low risk*	.54	.36−.73	Beta	[7]
***Outcome Parameters***				
***Life Years***				
*Benign Disease*	16.44	13.15-19.73	#	[20]
*Stage I*	16.32	13.06-16.44	#	[21]
*Stage II*	8.42	6.74-10.1	#	[21]
*Stage III, resectable*	5.04	4.03-6.05	#	[21]
*Stage III, unresectable*	3.84	3.07-4.61	#	[21]
*Stage IV*	3.34	2.67-3.84	#	[21]
***Procedural Costs***				
*Non-Surgical Biopsy*	$2,550	$2,040-$3,060	Gamma	CPT 31628, 32408 DRG 200, 201
*PET*	$1,480	$1,184-$1,776	Gamma	APC 5594
*Surveillance, growth*	$163	$130-$196	Gamma	CPT 71250
*Surveillance, stable*	$652	$326-$815	Gamma	CPT 71250
*AI-assistance*	$650	$100-$1,200	Gamma	CPT 0721T
*Wedge Resection*	$8,821	$7,057-$10,585	Gamma	DRG 166, 168
*Lobectomy*	$12,944	$10,355-$15,533	Gamma	DRG 163, 165
***Treatment Costs***				
*Adjuvant Chemotherapy (stage II, III) (ASP + 4.3%)*	$200,446	$160,357-$240,535	Gamma	Table 2
*Unresectable Stage III Chemotherapy (ASP + 4.3%)*	$193,426	$154,741-$232,111	Gamma	Table 2
*Stage IV Chemotherapy (ASP + 4.3%)*	$421,376	$337,101-$505,651	Gamma	Table 2
*Stage III XRT*	$166,933	$133,546-$200,320	Gamma	CPT 99205, 77263, 77334, 77293, 77301, 77338, 77300, 77387, 77427
***Unrelated Annual Medical Costs***				
*Age < 65*	$9,056	$7,245-$10,867	Gamma	[22]
*Age > 65*	$12,961	$10,369-$15,553	Gamma	[22]

*constrained by other model parameters; #not varied in probabilistic sensitivity analysis due to high confidence in value and stability of model with one-way sensitivity analyses; PSA: probabilistic sensitivity analysis; PET: Positron Emission Tomography; ASP: average sales price.

All malignant nodules that underwent surveillance were assumed to eventually grow on surveillance imaging and progress to lobectomy. Malignant nodules undergoing surveillance could experience a potential stage shift due to the delay in definitive management. Previously published models that investigated outcomes from delayed resection formed the basis for the stage shifting parameters [23].

For intermediate probability nodules, guidelines suggest further diagnostic evaluation via PET scan or non-surgical biopsy based on clinician and patient preference [5]. Following a non-diagnostic biopsy, patients in the model proceeded to either a diagnostic wedge resection or surveillance.

### Outcomes

The primary outcomes included discounted life years and the incremental cost-effectiveness ratio (ICER) of AI-supported nodule risk stratification compared to stratification performed by the clinician alone, calculated as cost per life year gained (LYG). We used a willingness to pay (WTP) threshold of $100,000/LYG and calculated life years associated with each outcome using the Declining Exponential Approximation of Life Expectancy (Table 1) [20, 21, 24]. For malignant diagnoses, we used life tables from the International Association for the Study of Lung Cancer Staging Project to calculate 5-year mortality rates associated with pathologic stage I, II, and III as well as clinical stage III and IV (for unresectable disease) [25]. Expected life years for the base case of patients with benign disease are from the life tables published in the National Vital Statistics Report [26]. For patients who suffered operative mortality, we assigned a life year value of zero.

Costs reflected a payer perspective using Centers for Medicare & Medicaid Services (CMS) reimbursement rates (Table 1) [22]. For procedures associated with an inpatient stay (wedge resection, lobectomy), we used diagnosis related groups (DRG) to capture all costs associated with the procedure [27]. For outpatient procedures, we estimated costs using CPT or ambulatory payment classification (APC) codes [22]. We obtained unrelated healthcare costs for each year of survival from the Medical Expenditure Panel Survey from the Agency for Healthcare Research and Quality [28].

As the impact of procedural morbidity on overall quality-adjusted life years would be minimal and temporary compared to survival differences for lung cancer, we incorporated procedural morbidity into the model via a weighted cost adjustment for the associated procedures (for details on cost weighting, see supplementary methods). We assumed the cost of procedural mortality was equivalent to the cost of the procedure with a major complication.

Anticancer medication costs are based on standard treatment regimens by stage outlined in the National Comprehensive Cancer Network (NCCN) guidelines for adenocarcinoma [29]. As lung cancer therapy is complex and becoming more highly individualized, we made assumptions in order to simply and accurately model costs of medical and radiation oncologic therapy by stage. In determining therapy costs, we considered malignant nodules as adenocarcinoma and eligible for anticancer therapy (chemotherapy, targeted therapy and/or immunotherapy) without targetable mutations. In calculating medication dosing, the base case represented the size of an average American male (200 pounds, 70 inches tall, with a body surface area of 2 m^2^). Additionally, while we understand that neoadjuvant therapy is now approved by the Food and Drug Administration and beginning to be incorporated in treatment for Stage IB to IIIA cancers at large tertiary centers, because its use is not yet widespread, we only included adjuvant anticancer treatment regimens [18]. Cost values for anticancer therapy are based on average sales price data plus a 4.3% markup that is standard for Medicare drug reimbursement [30]. Anticancer treatment regimens used for cost calculations by stage are outlined in Table 2 and were developed with thoracic medical oncologists in accordance with the NCCN guidelines to represent a standard regimen.

**Table 2:** Chemotherapeutic Regimen by Stage.

	Agent	Dose	Number of Infusions	Total Cost
**Stage I**	No adjuvant therapy	–	–	$0
**Stage II-IIIA**	Cisplatin	75 mg/m^2^	4	$101
	Pemetrexed	500 mg/m^2^	4	$8,617
	Pembrolizumab	200 mg	17	$188,418
	Total			$192,182
**Unresectable Stage III**	Carboplatin	AUC 2	6	$71
	Paclitaxel	45 mg/m^2^	6	$92
	Durvalumab	10 mg/kg	26	$185,289
	Total			$185,452
**Stage IV**				
PD-L1 ≥ 50%	Pembrolizumab	200 mg	35	$387,919
	Total			$387,919
PD-L1 < 50%	Carboplatin	AUC 5	4	$119
	Pemetrexed	500 mg/m^2^	35	$32,050
	Pembrolizumab	200 mg	35	$387,919
	Total			$420,088

In our model, clinicians administered radiation therapy exclusively for unresectable stage III tumors in combination with anticancer therapy, followed by consolidative durvalumab treatment. We calculated this cost using the sum of CPT codes necessary to perform intensity-modulated radiotherapy (IMRT). The base case was a medically operative candidate with a lesion anatomically amenable to resection. Therefore, if the malignancy qualified for resection from an oncologic standpoint, the patient would proceed with resection. Consequently, stereotactic body radiation therapy (SBRT) was not included in the model.

The model did not incorporate rates of local or distant disease recurrence after initial therapeutic failure (progression to stage IV disease after concurrent chemoradiotherapy for stage III disease), and hence any costs associated with treatment for recurrence after primary therapy were not accounted for.

### Sensitivity analysis

We performed a one-way sensitivity analysis on each parameter in the model, varying parameters by a minimum of ±20% from the base value. If the associated literature review demonstrated values with greater than ±20% variation, we incorporated the lower and upper bounds reported in the literature (Table 1). We varied all clinical decision probability events across a range of 0–1 to assess the model’s robustness under a wide range of clinical practice conditions. As FDG-PET has demonstrated decreased specificity in regions of endemic fungal lung disease, we conducted a two-way sensitivity analysis on the frequency of obtaining FDG-PET for intermediate-probability nodules and PET specificity [31]. We varied the frequency of PET use for intermediate-probability nodules widely from 0% to 100% to represent potential practice pattern variations and varied the specificity of PET from 49% to 81% to simulate both regions that are endemic and non-endemic, respectively, for fungal lung disease [31].

We conducted a probabilistic sensitivity analysis using second-order Monte-Carlo simulations by drawing 400 random parameter sets from prespecified distributions. We defined beta distributions for all probabilities and gamma distributions for all costs. We parameterized the mean of each distribution using the base case value and setting the standard deviation as half the range of the tested parameter in deterministic sensitivity analyses. This ensured that the entire parameter range tested in deterministic sensitivity analyses fell within two standard deviations. We evaluated the probability of AI-assistance being cost-effective using cost-effectiveness acceptability curves, varying the WTP between $0 and $200,000/LYG.

## Results

For the base case scenario, AI-supported pulmonary nodule risk stratification resulted in an increase of 0.03 life years as compared to clinician alone and an incremental cost of $114. With a 65% pre-test probability of malignancy, AI was cost-effective at a willingness to pay threshold (WTP) of $100,000/LYG with an incremental cost-effectiveness ratio (ICER) of $4,485/LYG (Table 3).

**Table 3:** Incremental Cost and Effectiveness of Clinician Compared to AI-Assisted Pulmonary Nodule Risk Stratification.

*Strategy*	*Cost (2021 USD)*	*Incremental Cost*	*Effectiveness (Life Years)*	*Incremental Effectiveness*	*ICER ($/life year)*
*Clinician*	$216,093	–	14.63	–	
*AI-Supported*	$216,207	$114	14.65	.03^*^	4,485

* Please note the incremental effectiveness represents unrounded model outputs and is.03, while the reported effectiveness (14.63 vs. 14.65 Life Years) is rounded to two decimals by convention.

### Sensitivity analyses

**Fig 2:**
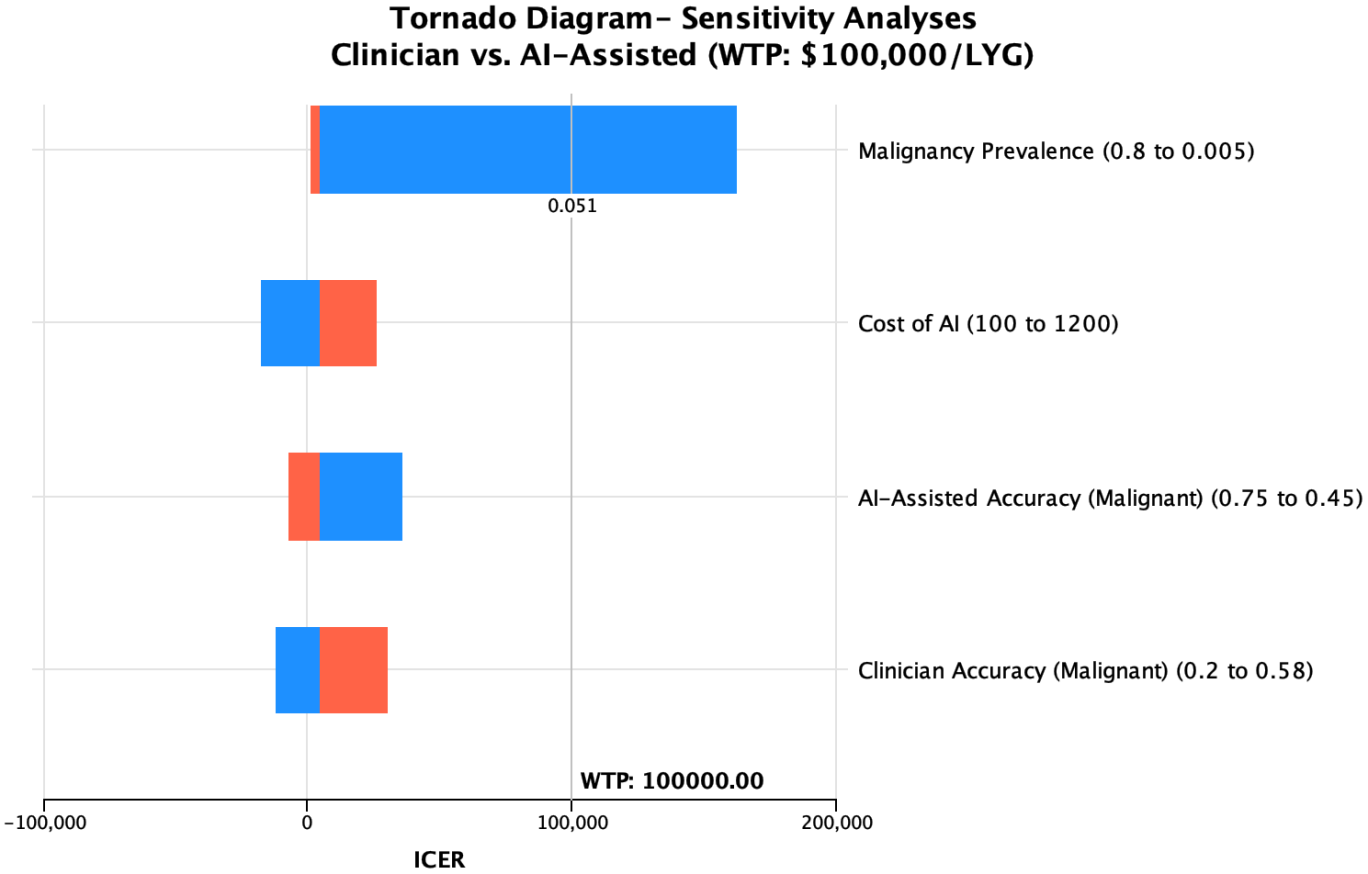
Tornado Diagram of Incremental Cost-Effectiveness Ratio Range of Four Most Influential Model Parameters. The tornado diagram demonstrates the incremental cost-effectiveness ratio (ICER) range for the 4 most influential parameters in one-way sensitivity analyses. Blue indicates the lower end of the varied parameter range and orange indicates the higher end of the range. The ICER exceeds the willingness-to-pay threshold only for malignancy prevalence when the prevalence is below.051. All other parameters remain cost-effective over the full tested range.

**Fig 3:**
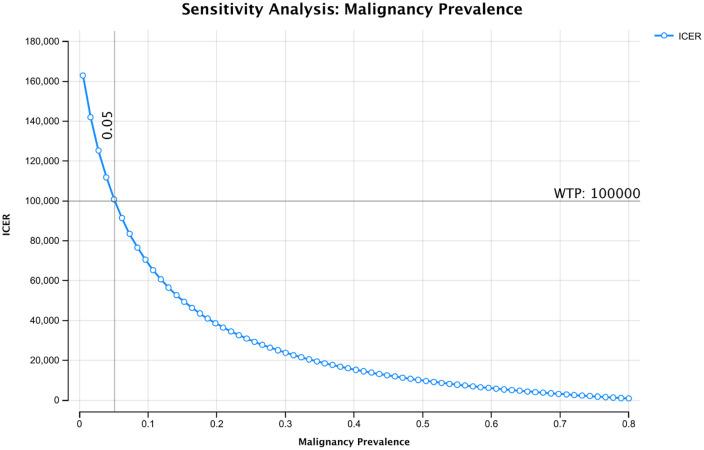
One-Way Sensitivity Analysis of Malignancy Prevalence. The x-axis shows the population malignancy prevalence, ranging from 0% on the left to 80% (0.8) on the right. The y-axis shows the incremental cost-effectiveness ratio (ICER) at the corresponding malignancy prevalence. The ICER exceeds the willingness-to-pay threshold when malignancy prevalence of the population is less than 5%.

A tornado diagram of the four most influential variables (malignancy prevalence, cost of AI, AI-assisted accuracy, and clinician accuracy) from the one-way sensitivity analyses conducted is shown in Fig 2. Notably, as the pre-test probability of malignancy was decreased, the ICER for AI-support increased and eventually exceeded the WTP threshold of $100,000/LYG when malignancy prevalence decreased below 5% (Fig 3). When a more conservative WTP threshold of $50,000/LYG was applied, AI-support remained cost-effective when malignancy probability was 15% or higher.

**Fig 4:**
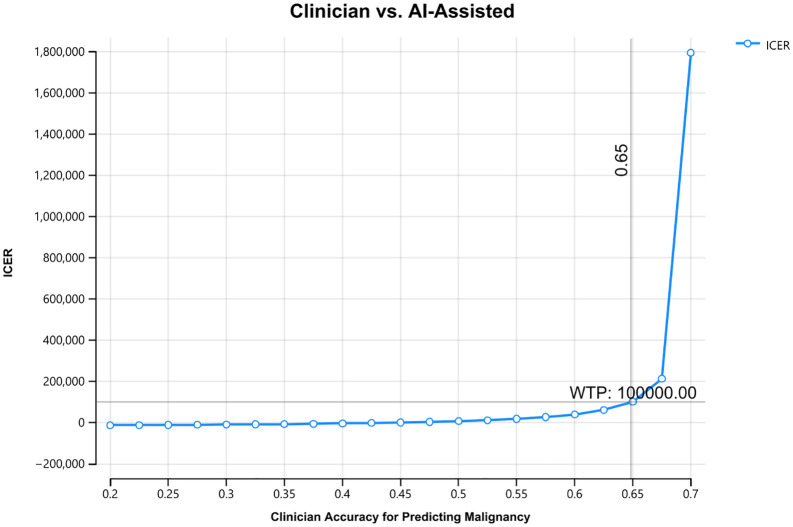
Sensitivity Analysis of Clinician Accuracy for Predicting Malignancy. The x-axis shows the probability a clinician correctly classifies a malignant nodule as high-risk. The y-axis shows the incremental cost-effectiveness ratio (ICER) corresponding to that probability. The ICER exceeds the willingness-to-pay threshold when the clinician accuracy for correctly identifying malignant nodules as high-risk exceeds 65%.

When varying clinician accuracy and holding AI-accuracy constant, AI-support became less cost-effective as clinician accuracy increased and eventually exceeded the WTP threshold of $100,000/LYG when the clinician correctly identified malignant nodules as high risk greater than 65% of the time (Fig 4). The reader study data used for performance parameters shows clinician accuracy of 47% in classifying malignant nodules as high risk [9].

AI-support was increasingly cost-effective as PET specificity decreased, though remained cost-effective at the WTP threshold of $100,000/LYG at all value combinations analyzed in the two-way sensitivity analysis.

**Fig 5:**
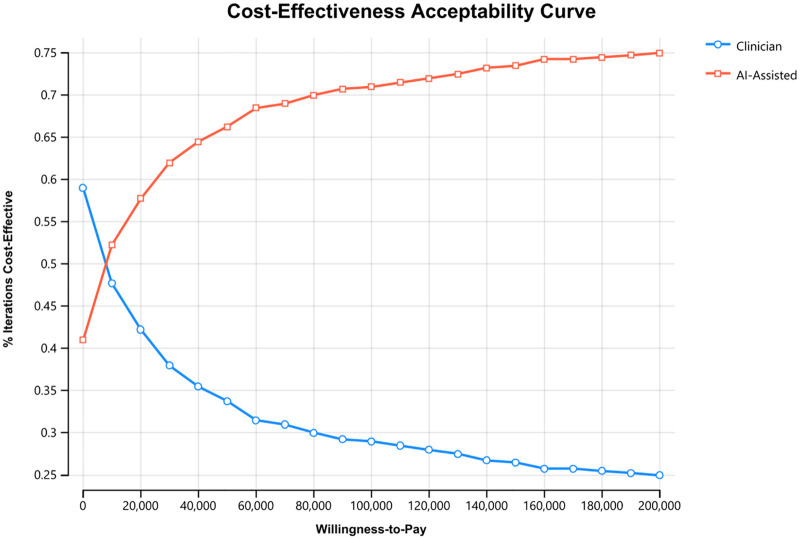
Probabilistic Sensitivity Analysis: Cost-Effectiveness Acceptability Curve. The x-axis represents varying willingness-to-pay thresholds. The y-axis represents the percentage of iterations of the model that resulted in either strategy being cost-effective at the corresponding willingness-to-pay. The curves cross at $4,058, the willingness-to-pay threshold where 50% of the iterations were cost-effective for each strategy. At the base case willingness-to-pay of $100,000/life year gained, AI-assistance is cost-effective in 71% of iterations.

In the probabilistic sensitivity analysis, the cost-effectiveness acceptability curves demonstrated that AI-assistance was cost-effective in greater than 50% of the 400 iterations performed when the WTP threshold was greater than $4,058 (Fig 5). AI-assistance had a 71% probability of being cost-effective at the base-case WTP threshold ($100,000/LYG).

## Discussion

As AI is increasingly woven into medical decision-making and these platforms come to market, it is important to evaluate their cost-effectiveness. This study suggests that AI-supported IPN risk stratification, at the observed accuracies from the previously published 12-reader LCP Score study, may be cost-effective in populations with an underlying malignancy rate of 5% or greater [9]. Reported malignancy rates amongst nodules detected in a screening population are estimated around 5% [32]. Among all incidentally-discovered pulmonary nodules, malignancy prevalence is reported to be only 3%, however incidentally discovered nodules in patients with a history of tobacco use have a higher prevalence, reported at 5.5%, and nodules measuring larger than 1 cm have a 12–15% malignancy risk [33]. Malignancy prevalence in dedicated pulmonary nodule and thoracic surgery clinics is reported to be even higher, approaching 60–80% [11]. In this context, AI support would be an appropriate and cost-effective method for nodule risk stratification, though further investigation of the potential cost-effectiveness in a general screening population with malignancy prevalence near the threshold of 5% is warranted due to the greater risk of false positive findings. Additionally, real-world guideline-driven practices and radiologist expertise for thoracic screening studies vary worldwide which may also limit the potential benefit of these tools. Moreover, the racial and ethnic makeup of global populations may not be fully represented in the training cohorts used to build this, and other, AI tools.

Though this model was robustly tested in both deterministic and probabilistic sensitivity analyses which all returned similar results, supporting the conclusion of the cost-effectiveness of AI support in pulmonary nodule risk estimation, this study has limitations. One limitation of the study is that we assume guideline-based diagnostic evaluation and management and therefore the model is not reflective of true clinical practice at this time. Significant variation in management of indeterminate pulmonary nodules exists and clinical practice has been demonstrated to deviate significantly from guideline-based care [34]. This model was built to resemble guideline-based care as closely as possible. We acknowledge that real clinical practice patterns vary, particularly as guidelines and evidence in lung cancer treatment and diagnosis continue to evolve [35]. For example, emerging data demonstrating the oncologic equivalence of sublobar resections for early-stage lung cancers is likely to shift practice away from routine lobectomy for lung cancer [36]. Moreover, the surveillance branch in our model is limited in that it did not include potential nonoperative management,competing mortality, or loss to follow-up. Our base case patient was a good operative candidate and the model was intentionally structured to focus on differences in diagnostic workup as opposed to variable long-term treatment adherence. For this reason, malignant nodules detected during surveillance were assumed to eventually undergo definitive surgical management. Ultimately, it is unknown at this time how clinicians would interact with AI-based support tools and how this might affect their clinical decision-making. The cost-effectiveness, or effectiveness at all, of AI-supported risk stratification hinges on a resulting change in clinical management. If no changes occur in clinical management based on improvements in risk stratification, then the accuracy of the risk estimation is inconsequential to patient outcomes. This practice pattern variation would be best evaluated in a future pragmatic trial.

An additional limitation is that costs associated with treatment for recurrence after primary therapy were not incorporated into the model due to the high variation of these costs. As such, the model likely underestimates the cost of delayed diagnosis of early-stage malignancy due to inaccurate risk estimation. Incorporating these downstream costs is likely to make the use of AI support increasingly cost-effective due to increased cost penalization for late-stage disease treatment. Additionally, it should be noted that this model was created from the payer perspective, not the institutional perspective, and as such, does not include costs related to an institutional contract for use of an AI platform.

The cost-effectiveness of AI-supported risk stratification is heavily dependent on both clinician and AI accuracy. Another limitation of this study is that the accuracy of both the clinician and the AI-supported stratification are based on a single study based on 12 readers analyzing 300 nodules [9]. This cohort was not a consecutive clinical cohort and was cancer-enriched, albeit not compared to the 65% prevalence assumed here. A direct comparison of clinician alone and AI-supported nodule stratification in a consecutive clinical cohort is not currently available. It is possible that both clinician and AI performance would differ in varying clinical settings, but at this time, it is challenging to know whether their accuracies would change relative to each other.

In settings with particularly high clinician accuracy in lung nodule risk stratification, the cost-effectiveness of AI-support decreases as the accuracy of clinician alone and AI-supported stratification converge. Conversely, in clinical settings with lower clinician accuracy, the cost-effectiveness of AI-supported risk stratification would increase as the accuracies of the two methods diverge. Therefore, even when applied to the same patient population, the cost-effectiveness of AI-support will vary by institution according to clinician accuracy. Reports of clinician accuracy in identifying cancerous nodules are limited, making it challenging to distinguish which clinical settings are appropriate for the cost-effective utilization of AI. In the reader study used for parameterization of risk stratification in this model, the clinicians accurately identified malignant nodules as high cancer probability 47% of the time, however, another study conducted by Tanner and colleagues demonstrates a higher rate of malignant nodule identification among thoracic surgeons and pulmonologists [9,37]. Though there are limited data on this topic, in settings with limited access to sub-specialty trained pulmonologists and thoracic surgeons, AI is likely to accelerate the process of referral of patients with likely malignant pathology to a more specialized center.

## Conclusion

AI-assisted pulmonary nodule risk estimation is cost-effective in most common clinical settings where the malignancy prevalence is at least 5%. With high rates of lung resections performed on benign disease and the necessity of rapid identification of lung cancer to initiate treatment in its earliest, most treatable phase, the need for accurate lung cancer risk estimation has never been greater. AI offers promise of improved risk estimation of lung nodules at reasonable cost and if used in a more automated process, could reduce some of the clinical burden on physicians to manually perform this risk stratification as IPNs are increasingly identified. A clinical trial should be considered to test the risk estimation improvement of AI over the clinician more robustly in clinical settings with varying nodule size, nodule shape, nodule location, cancer prevalence, and rates of fungal lung disease. As seen in other areas of medicine, AI cannot replace the clinician, but AI-assistance could improve their ability to evaluate and treat their patients in a cost-effective manner.

## Supporting information

S1 File Full variable definitions – Excel file. Excel file containing the complete list of variable definitions included in the model.
(XLSX)
